# Expanding the Clinical Spectrum of VEXAS (Vacuoles, E1 Enzyme, X-linked, Autoinflammatory, Somatic) Syndrome: The First Report of Histologically Confirmed Neutrophilic Colitis With Rare Muscular and Pancreatic Involvement

**DOI:** 10.7759/cureus.101715

**Published:** 2026-01-16

**Authors:** Sarah El Tahech, Hussein Nassereddine, Sara Moukheiber, Georges Maalouly

**Affiliations:** 1 Departement of Internal Medicine and Clinical Immunology, Saint Joseph University of Beirut, Beirut, LBN; 2 Departement of Anatomopathology, Saint Joseph University of Beirut, Beirut, LBN

**Keywords:** colonic histopathology, macrocytic anaemia, masseter myositis, micro-erosion, neutrophilic infiltrate, orbital myositis, pancreatic enhancement, pet-ct scan, venous thrombo-embolism, vexas syndrome

## Abstract

VEXAS (Vacuoles, E1 Enzyme, X-linked, Autoinflammatory, Somatic) syndrome is a rare, adult-onset autoinflammatory disorder caused by somatic mutations in the *UBA1* gene, most commonly affecting older men and associated with systemic inflammation and hematologic abnormalities. Although hematologic, cutaneous, and cartilaginous manifestations are well recognized, involvement of other organs, including muscle, pancreas, and colon, has been infrequently reported. We report a case of a 77-year-old male who initially presented with orbital inflammation, facial myositis, and laryngeal edema, followed by extensive superficial and deep venous thromboses. His clinical course subsequently included recurrent fevers and gastrointestinal bleeding. Laboratory evaluation revealed macrocytic anemia and elevated inflammatory markers. Colonoscopy demonstrated findings consistent with ulcerative colitis, and histopathology showed dense neutrophilic infiltration with microthrombi and fibrinoid necrosis. Positron emission tomography scanning revealed hypermetabolic uptake in the pancreatic head, lymph nodes, and bone marrow. Genetic testing confirmed a somatic *UBA1* mutation, thereby establishing the diagnosis of VEXAS syndrome.

This case report broadens the recognized clinical spectrum of VEXAS syndrome by combining three rarely described manifestations - facial (orbital/masseter) myositis, pancreatic hypermetabolism, and neutrophilic colitis with vascular injury. To our knowledge, this is the first report to present detailed colonic histopathologic findings in VEXAS syndrome. VEXAS syndrome remains underrecognized, particularly in the presence of atypical organ involvement. Awareness of uncommon features- such as colonic neutrophilic vasculitis or facial myositis - may prompt earlier clinical suspicion, facilitate targeted genetic testing, and support timely, appropriate management. Further systematic studies are needed to delineate the full clinical spectrum, histopathologic features, and optimal therapeutic strategies for VEXAS.

## Introduction

VEXAS syndrome (Vacuoles, E1 Enzyme, X-linked, Autoinflammatory, Somatic) is a recently recognized adult-onset autoinflammatory disease caused by a somatic mutation in the *UBA1* gene, located on the X chromosome [1-3]. This monogenic disorder was first described in December 2020 by Beck et al. [3]. It predominantly occurs in older men, with a median age at diagnosis of approximately 67 years [1], and presents with a broad spectrum of systemic inflammatory symptoms associated with hematologic abnormalities [2,4].

The most frequent manifestation is hematologic, with macrocytic anemia present in nearly all reported cases [2,4], followed by systemic symptoms such as fever and weight loss (reported in 65-100% of cases) [2]. The third most frequently reported manifestation involves skin lesions, which typically present as painful papules and nodules [1], with histopathology showing neutrophilic dermatosis in 83% of cases [1,2,5,6]. Other manifestations include vasculitis, thromboembolic events (10-56%) [2,4], pulmonary nodules (49%) [1,2], ophthalmological involvement (40% of cases, with various presentations such as episcleritis, scleritis, uveitis) [1], and arthralgias [7]. At presentation, the majority of patients show elevated inflammatory markers such as C-reactive protein (CRP) and erythrocyte sedimentation rate (ESR) [2], confirming a systemic inflammatory state [3].

However, given the recent discovery of this syndrome, new manifestations are still being identified. While systemic involvement is well-recognized, muscular, pancreatic, and gastrointestinal presentations have only been rarely reported. Only a few cases of VEXAS syndrome have described muscle involvement [8,9] or pancreatic findings on imaging [10,11]. To our knowledge, histopathologic findings involving the gastrointestinal tract have not previously been reported. We present a case of genetically confirmed VEXAS syndrome with three rarely described clinical manifestations: orbital and masseter myositis, pancreatic hypermetabolism on PET imaging, and histologically confirmed neutrophilic colitis with vasculitic features. These findings expand the known clinical spectrum of VEXAS and highlight the importance of recognizing atypical organ involvement in affected patients.

## Case presentation

**Figure 1:**
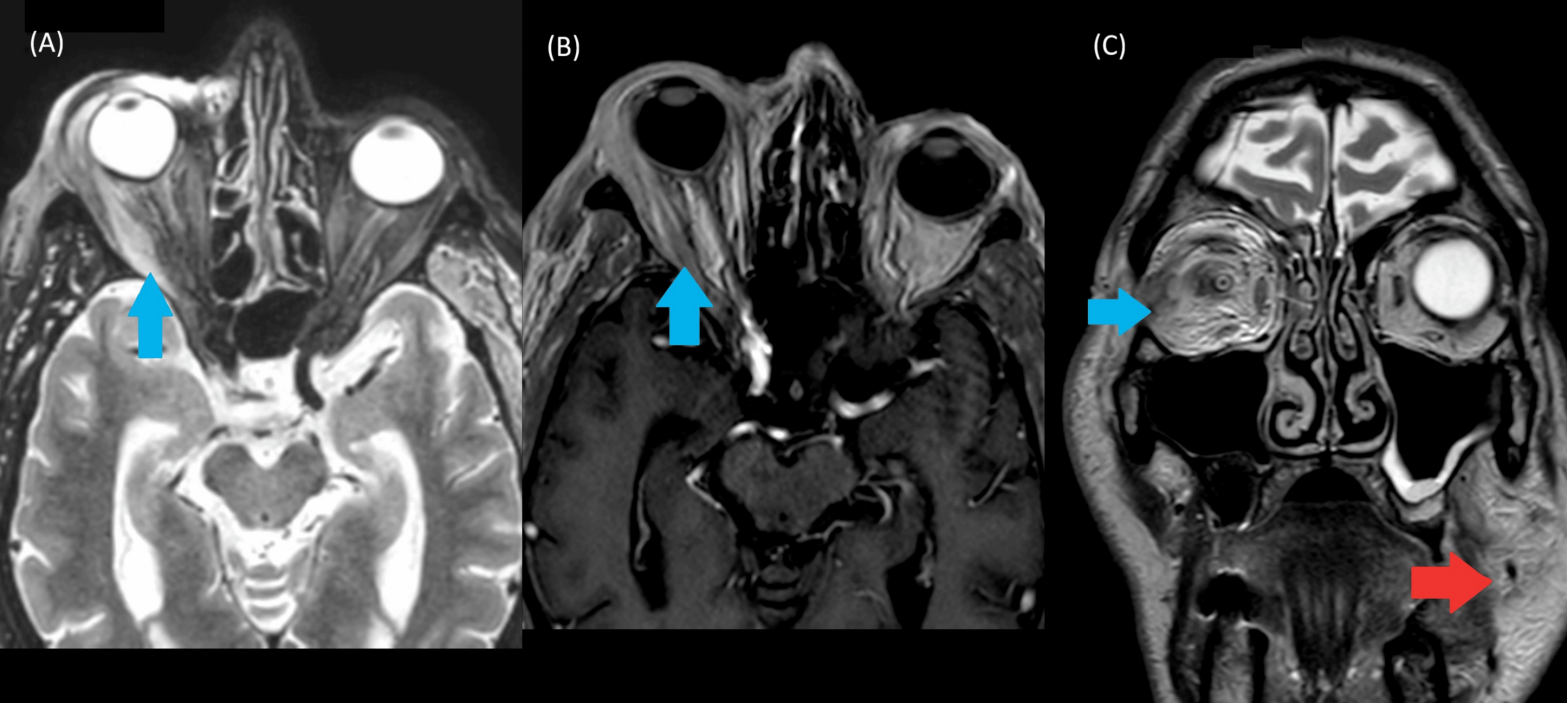
Cerebral and orbital MRI (A) Orbital MRI (axial T2-weighted and STIR-long TE sequences) demonstrating diffuse hyperintense signal and enlargement of the extraocular muscles (blue arrow), consistent with active inflammatory edema. (B) Orbital MRI (axial T1 post-gadolinium) showing diffuse enhancement and mild enlargement of the extraocular muscles (blue arrow), compatible with inflammatory myositis. (C) Cerebral MRI (coronal STIR) showing diffuse hyperintensity and enlargement of the extraocular muscles (blue arrow), reflecting active inflammatory edema, with concomitant edema of the masseter muscles (red arrow) MRI: magnetic resonance imaging; STIR: short tau inversion recovery

The patient was a 77-year-old male with a history of prostatic adenocarcinoma treated with prostatectomy 10 years prior and without other known comorbidities. He presented with periorbital edema, exophthalmos, diplopia, and conjunctival redness. These symptoms partially and spontaneously improved over the ensuing weeks. During the etiologic workup, the patient developed odynophagia and dysphonia. An urgent nasofibroscopy revealed edema of the lingual surface of the epiglottis, which prompted immediate corticosteroid treatment in the ENT department. An MRI revealed right-sided exophthalmia associated with optic nerve stretching, diffuse edema of the extraocular muscles, and myositis affecting the left masseter, temporalis, and pterygoid muscles (Figure 1). The edema resolved within 48 hours following corticosteroid therapy, and the patient was discharged without continued corticosteroid treatment to allow further diagnostic investigations.

One month later, the patient developed both superficial and deep vein thromboses in all four limbs, together with myositis of the forearm muscles. These findings were confirmed by venous Doppler ultrasound and soft tissue ultrasonography, respectively, with spontaneous resolution of the myositis after five days. This was followed by recurrent fever (peaking at 39 °C) and melena. Laboratory tests revealed macrocytic anemia with hemoglobin (Hb) 8 g/dL (normal range: 13-17 g/dL) and a mean corpuscular volume (MCV) of 101 fL (normal range: 78-98 fL), normal platelets level (256,000 ×10⁹/L (normal range: 150-450 ×10⁹/L), a markedly elevated ESR of 139 mm/h (normal range: 0-10 mm/h), a CRP level of 109 mg/L (normal range <5 mg/L), and a low transferrin saturation of 10% (normal range: 19-39%). Serum folate, vitamin B12, and thyroid-stimulating hormone levels were within the normal range (Table 1). 

**Table 1:** Laboratory results TSH: thyroid-stimulating hormone; ANA: antinuclear antibody; ANCA: antineutrophil cytoplasmic antibodies; CPK: creatine phosphokinase; IgG4: immunoglobulin G4; PCR: polymerase chain reaction

Laboratory test	Result	Reference range	Units
Folat (vitamin B9)	5.6	3–20	ng/mL
Vitamin B12	267	200–900	pg/mL
TSH	0.83	0.4–4.0	mIU/L
Fibrinogen	5	2.0-4.0	g/L
Peripheral blood smear	anisocytosis, poikilocytosis, and acantocytes		
ANA	Negative	Negative	–
ANA blot	Negative	Negative	–
Rheumatoid factor	Negative	Negative	–
C-ANCA	<2	<20	RU/mL
P-ANCA	5.25	<20	RU/mL
C3	127	90–180	mg/dL
C4	36	10–40	mg/dL
Anti-cardiolipin	2.7	<12	RU/mL
Anti-β2-glycoprotein	5.1	<20	RU/mL
Lupus anticoagulant	Negative	Negative	–
Serum protein electrophoresis	Inflammatory pattern	Normal	–
CPK	42	55–170	U/L
IgG4	23	3–201	mg/dL
Myositis antibody panel (Mi2, TIF1, MDA5, NXP2, SAE, KU, PMSCL, JO1, SRP, PL7, PL12, EJ, OJ)	Negative	Negative	–
Multiplex intestinal PCR and Clostridium PCR	Negative	Negative	–

**Figure 2:**
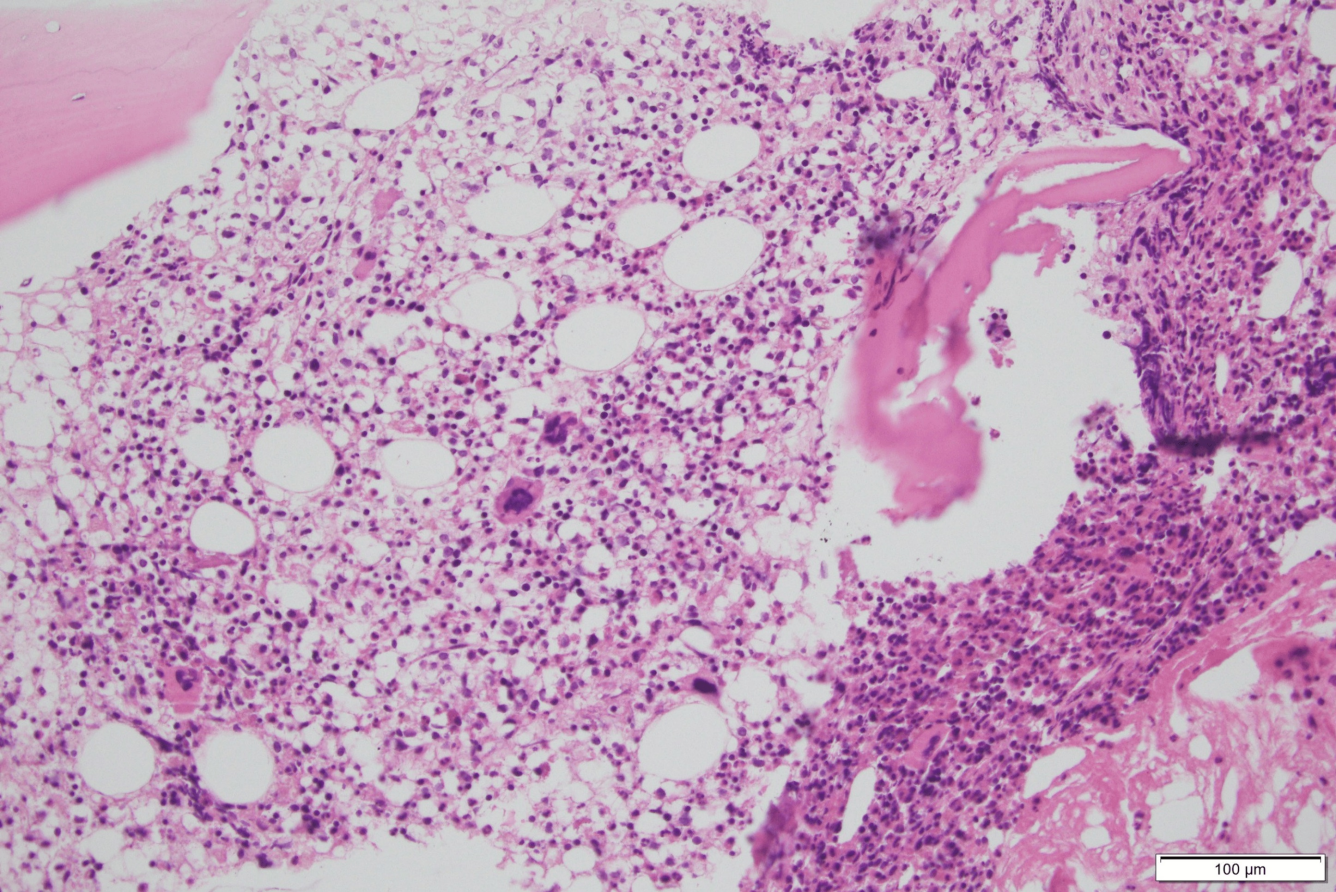
Bone marrow histopathology showing hypercellular marrow with granulocytic predominance The marrow cellularity is increased with a decreased myeloid to erythroid ratio, granulocytic predominance, and scattered megakaryocytes in various stages of maturation and unremarkable morphology (H&E x20) H&E: hematoxylin and eosin

**Figure 3:**
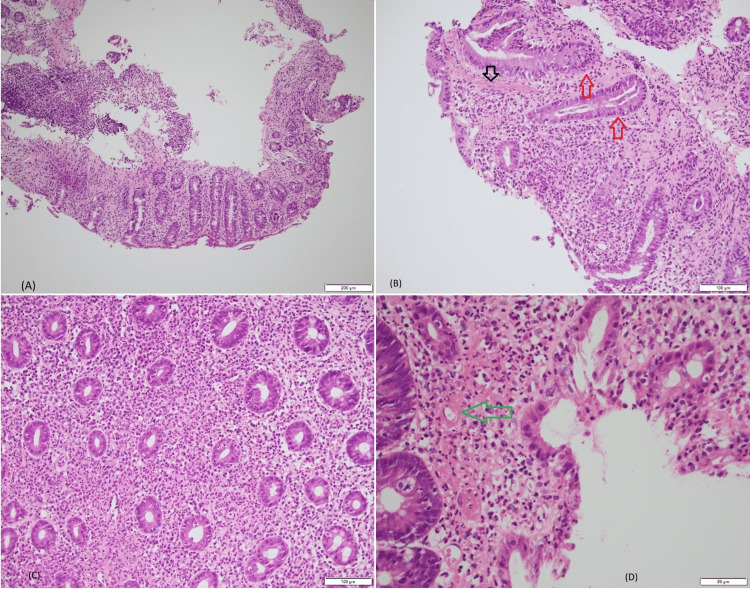
Histopathological findings from colon biopsies (A) Focal superficial epithelial denudation with crypt regenerative changes and lamina propria hyalinization, consistent with an ischemic-like pattern (H&E x10). (B) The colonic mucosa shows regenerative epithelial change (red arrow) characterized by crypt elongation, loss of mucin secretion with nuclear enlargement and hyperchromasia, nuclear stratification limited to the basal portion of the crypts, and preserved nuclear polarity. No dysplasia is identified. Microerosions, dense infiltrate of neutrophils in the lamina propria, and microvascular thrombi (black arrow) are also identified (H&E x20). (C) Dense mixed inflammatory infiltrate with neutrophilic predominance in the lamina propria, neutrophilic exocytosis within crypt epithelium, and crypt regeneration (H&E x20). (D) Prominent neutrophilic infiltrate with epithelial exocytosis and crypt regeneration at higher magnification, and fibrinoid deposits around blood vessels (green arrow) (H&E x40) H&E: hematoxylin and eosin

Bone marrow biopsy revealed hypercellular marrow with granulocytic predominance and no evidence of vacuolization (Figure 2). Upper and lower endoscopies demonstrated colitis with small superficial ulcerations and extensive whitish plaques extending from the rectum to the left colon, associated with patchy erythematous mucosa. Histopathological analysis revealed diffuse exudative neutrophilic inflammation with microerosions, superficial microthrombi, and fibrinoid necrosis (Figure 3), suggesting an inflammatory and vasculitic process, with no evidence of granulomas.

**Figure 4:**
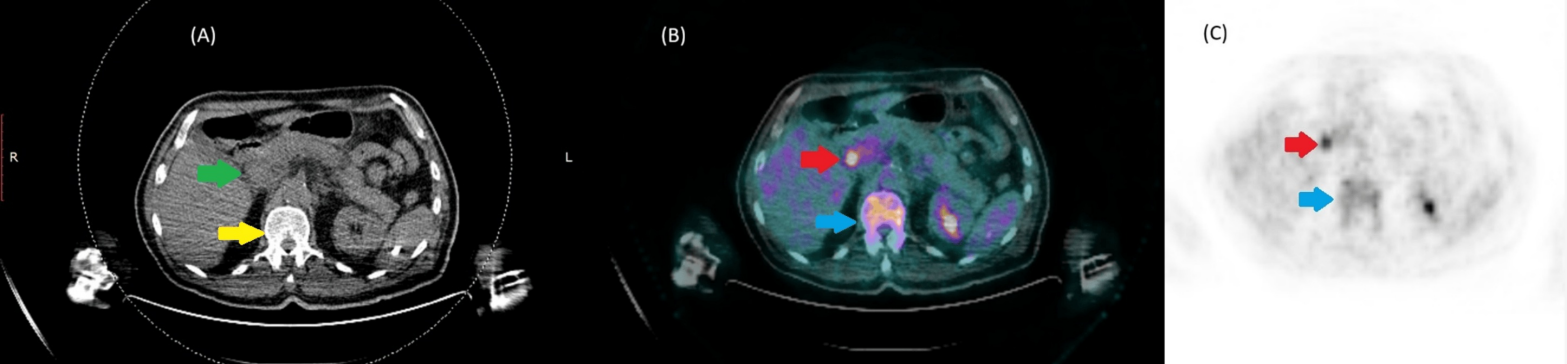
Axial PET-CT images of the abdomen showing hyper metabolism in the pancreas and bone marrow (A) Axial non-contrast CT image identifying the anatomical structures, including the head of the pancreas (green arrow) and axial bone marrow (yellow arrow), without focal mass or structural abnormality. (B) Fused PET/CT image demonstrating focus of increased FDG uptake at the level of the pancreatic head with SUVmax 8.3. (red arrow) and diffuse hypermetabolic activity within the bone marrow (blue arrow), suggestive of active inflammatory or hematopoietic involvement. (C) PET-only image highlighting the corresponding metabolic activity without anatomical detail: head of the pancreas (red arrow) and bone marrow (blue arrow) PET-CT: positron emission tomography-computed tomography; FDG: fluorodeoxyglucose; SUVmax: maximum standardized uptake value

A comprehensive diagnostic work-up for infectious etiologies, connective tissue diseases, vasculitis, myositis, and antiphospholipid syndrome was negative (Table 1). Thoraco-abdominopelvic CT scan demonstrated nodular pleural thickening with subpleural alveolar consolidation. Given this constellation of findings, a PET-CT scan was performed to rule out malignancy. It showed hypermetabolic activity in the pancreatic head, cervical and mesenteric lymphadenopathy, and increased uptake in the bone marrow (Figure 4).

**Figure 5:**
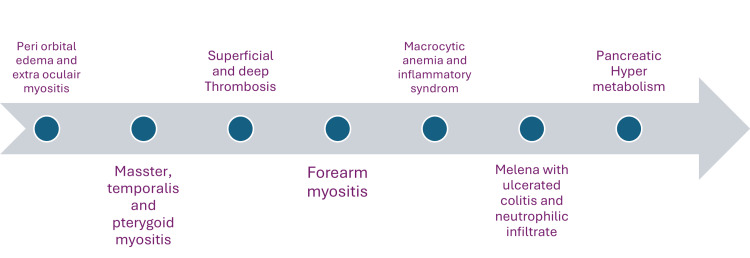
Timeline summarizing the chronological clinical manifestations in the patient The diagram illustrates the sequential development of multisystem involvement, beginning with periorbital edema and extraocular myositis, followed by facial and forearm myositis, extensive superficial and deep venous thromboses, macrocytic anemia with systemic inflammation, gastrointestinal bleeding due to neutrophilic colitis, and subsequent identification of pancreatic hypermetabolism, ultimately leading to the diagnosis of VEXAS syndrome VEXAS: Vacuoles, E1 Enzyme, X-linked, Autoinflammatory, Somatic

Endoscopic ultrasound of the pancreas showed normal parenchyma. Given the presence of macrocytosis and multi-organ inflammatory involvement, a genetic test was performed for VEXAS syndrome, revealing the presence of a somatic mutation in the *UBA1* gene, thereby establishing the diagnosis. Treatment was initiated with corticosteroids at 0.5 mg/kg/day (40 mg/day) in combination with methotrexate at 0.3 mg/kg/week (25 mg weekly). At the three-month follow-up, the patient showed marked clinical improvement, with resolution of the inflammatory syndrome (CRP: 5 mg/L), an increase in Hb to 10.5 g/dL, and no further episodes of myositis or thrombophlebitis. Corticosteroids were progressively tapered, and close follow-up was maintained to monitor for relapse. Figure 5 depicts a timeline of the chronological clinical manifestations in the patient.

## Discussion

This case represents a rare and complex form of VEXAS syndrome. Our patient presented with multiple inflammatory symptoms that evolved over time. Initially, he developed periorbital edema, exophthalmos, diplopia, and conjunctival redness. These symptoms partially improved spontaneously but were followed by epiglottic swelling, accompanied by myositis affecting the extraocular muscles, as well as the left masseter, temporalis, and pterygoid muscles. These initial manifestations were non-specific and primarily suggestive of an infiltrative process. Over time, he developed multisystemic symptoms, including macrocytic anemia, fever, gastrointestinal bleeding (melena), widespread venous thromboembolism, and pancreatic abnormalities - illustrating a complex association between inflammatory and hematological manifestations. In light of these findings, genetic testing was pursued, ultimately confirming the diagnosis of VEXAS syndrome.

VEXAS syndrome is a recently described adult-onset autoinflammatory disease, primarily affecting older men, characterized by systemic inflammation, hematologic abnormalities (especially macrocytic anemia), and somatic *UBA1* mutations in the *UBA1* gene located on the X chromosome. Clinical manifestations are heterogeneous but commonly include recurrent fevers, chondritis, vasculitis, and skin involvement [1,2]. Although bone marrow vacuolization of myeloid and erythroid precursors is considered a hallmark [2,3], its absence does not exclude the diagnosis [12]. In our patient, bone marrow biopsy showed hypercellularity with granulocytic predominance but no vacuoles (Figure 1). Similar findings have been reported in genetically confirmed cases, indicating that vacuolization may be focal, transient, or absent depending on disease stage, sampling variability, or prior corticosteroid exposure [4,12]. Also, marrow vacuoles are not specific and may occur in myelodysplastic syndromes or metabolic/toxic conditions [4]. Hence, *UBA1* mutation analysis remains the diagnostic cornerstone in clinically suspected cases [2].

The manifestations in our patient, including recurrent fevers, weight loss, thromboses, and macrocytic anemia, reflect the classic inflammatory and hematological features of VEXAS syndrome and align with patterns reported in the literature [2]. However, the initial presentation and additional rare features underscore the atypical nature of this case, as facial myositis, pancreatic hypermetabolism, and neutrophilic colitis are only rarely reported. Regarding gastrointestinal involvement, our patient exhibited two uncommon features. Anemia combined with low transferrin saturation and melena strongly suggested an underlying gastric or colonic source. In a retrospective study of 12 patients with VEXAS syndrome, only two had colitis or intestinal inflammation, highlighting the rarity of gastrointestinal involvement [11]. Abdominal pain is uncommon but has been reported as a presenting or associated symptom [13]. Although colitis has been described in case reports, gastrointestinal bleeding is exceedingly rare, occurring in only 0.9% of cases [1].

Interestingly, to our knowledge, colonic histopathology in VEXAS syndrome has not been previously reported in the literature. To support this observation, a focused review of the literature was conducted using PubMed and Google Scholar, employing combinations of the terms “VEXAS syndrome,” “colon,” “colitis,” “histopathology,” and “anatomopathology.” This research failed to identify any published cases reporting histologically documented colonic involvement in VEXAS syndrome. Although gastrointestinal manifestations have occasionally been described, detailed colonic histopathological findings are absent in the existing literature.

In our case, histologic examination of endoscopic biopsies revealed diffuse acute inflammation characterized by a dense neutrophilic infiltrate and focal microerosions. The mucosa demonstrated regenerative epithelial changes, along with vascular congestion and stromal edema. Superficial microthrombi and fibrin deposits around blood vessels were also identified. These findings, illustrated in Figure 2, suggest an ischemic-like pattern of colitis, prompting a broad differential diagnosis. While primary ischemic colitis due to vascular insufficiency is a consideration, similar histology may occur in vasculitic, infectious, autoimmune, or drug-induced conditions [14]. The absence of granulomas or viral inclusions makes mycobacterial or viral etiologies less likely. Likewise, negative special stains further argue against the presence of fungal infection.

The lack of crypt architectural distortion makes chronic inflammatory bowel disease less probable. However, the presence of microthrombi and perivascular fibrin deposition raises suspicion for ischemic injury or thrombotic microangiopathy, and may also suggest vasculitis. Given the clinical presentation, this ischemic-like injury may represent a manifestation of VEXAS syndrome, a disorder characterized by endothelial dysfunction and a prothrombotic state that can lead to vascular and ischemic injury in multiple organs [11,15]. Notably, vasculitis has been increasingly recognized as part of the VEXAS spectrum, affecting up to one-quarter of patients [15,16].

Small-vessel involvement is most frequently reported, particularly cutaneous leukocytoclastic vasculitis [17], while medium-vessel disease is less common [17], and large-vessel inflammation occurs only rarely, sometimes resembling giant cell arteritis [3,18]. Similarly, just as vasculitis can appear as a systemic feature of VEXAS, colonic wall involvement may represent another manifestation of the syndrome, as suggested by our findings. Furthermore, the neutrophilic infiltrate observed in the colon mirrors patterns described in skin [5] and muscle biopsies [19] from patients with VEXAS, supporting the idea that neutrophilic infiltrate may be a pathognomonic feature of the disease in addition to inflammatory and hematologic manifestations.

Regarding the pancreatic findings, our patient demonstrated increased FDG uptake in the pancreatic head on PET imaging. According to the literature, only two other cases have reported pancreatic hypermetabolism, with infection and malignancy ruled out [10,11]. In both instances, the reduction in FDG uptake following glucocorticoid therapy supported an inflammatory cause. Additionally, one case of pancreatitis was reported in a 78-year-old male presenting with fatigue, nausea, and abdominal pain. Elevated lipase levels, CT scan findings, and PET hypermetabolism confirmed pancreatic inflammation. Following the exclusion of other causes, *UBA1* mutation testing confirmed the diagnosis of VEXAS. These findings raise the possibility that certain cases previously labeled as idiopathic pancreatitis may, in fact, represent underrecognized manifestations of VEXAS syndrome.

Lastly, our patient experienced multifocal myositis in atypical regions. While most reported cases of myositis in VEXAS syndrome involve the limbs, a retrospective study based on the international AIDA registry (n=59) identified orbital myositis in only two patients (3.4%) [8], diagnosed using ultrasound and MRI. Another report described a 57-year-old man with both orbital and lower limb myositis. MRI revealed bilateral intramuscular and fascial edema, particularly in the vastus lateralis and medialis. Muscle biopsy demonstrated necrotizing myopathy with CD68-positive macrophage infiltration, and two previously unreported *UBA1* mutations were identified [20]. Myositis of the masticatory muscles is even rarer, with only one published case of a 68-year-old man presenting with painful trismus. Imaging revealed inflammation of the masseter and pterygoid muscles, consistent with masticatory myositis. Genetic testing confirmed the presence of a rare somatic *UBA1* mutation [9]. Taken together, these findings make our case an exceptional example of facial myositis, involving the orbital, masseter, and pterygoid muscles, an exceedingly rare presentation of VEXAS syndrome.

This case, therefore, represents a rare and complex form of VEXAS syndrome. In addition to hallmark features such as macrocytosis, fever, and thromboses, he exhibited uncommon and underrecognized manifestations. Notably, no previously published case has reported micro-ulcerated neutrophilic colitis with vasculitic features. This neutrophilic colonic involvement may represent the intestinal counterpart of the neutrophilic dermatologic and myopathic manifestations commonly seen in this syndrome. Such a histopathological signature, combining neutrophilic infiltration with vascular lesions, could serve as a diagnostic clue when present. Additionally, masseter muscle myositis has only been reported once in the literature, in a case presenting with trismus, while orbital myositis has been reported in only two other cases. Pancreatic hypermetabolism observed on PET scan has been described in only two prior reports.

## Conclusions

VEXAS syndrome remains an underrecognized and evolving clinical entity. This report illustrates the diagnostic complexity associated with the condition and emphasizes the need for maintaining a high index of suspicion, particularly in elderly patients presenting with macrocytosis and diverse inflammatory features. Recognizing atypical organ involvement, such as colonic neutrophilic vasculitis or unusual myositis patterns, may aid in prompt diagnosis and appropriate genetic testing.
